# Investigating Stress Response during Vaginal Delivery and Elective Cesarean Section through Assessment of Levels of Cortisol, Interleukin 6 (IL-6), Growth Hormone (GH) and Insulin-Like Growth Factor 1 (IGF-1)

**DOI:** 10.3390/jcm8081112

**Published:** 2019-07-27

**Authors:** Nikolaos Kiriakopoulos, Sokratis Grigoriadis, Evangelos Maziotis, Anastasios Philippou, Anna Rapani, Polina Giannelou, Petroula Tsioulou, Konstantinos Sfakianoudis, Adamantia Kontogeorgi, Panagiotis Bakas, George Mastorakos, Michael Koutsilieris, Mara Simopoulou

**Affiliations:** 1 Department of Physiology, Medical School, National and Kapodistrian University of Athens, 75 Mikras Asias, 11527 Athens, Greece; 2 Fifth Department of Obstetrics and Gynecology, General Maternal Hospital of Athens “Elena Venizelou”, 2 Elenas Venizelou Square, 11527 Athens, Greece; 3 Unit of Endocrinology, Diabetes mellitus and Metabolism, 2nd Department of Obstetrics and Gynecology, Aretaieion Hospital, Medical School, National and Kapodistrian University of Athens, 76 Vasilissis Sofias Avenue, 11527 Athens, Greece; 4 Centre for Human Reproduction, Genesis Athens Clinic, 14-16 Papanikoli, Chalandri, 11527 Athens, Greece; 5 Assisted Reproduction Unit, 2nd Department of Obstetrics and Gynecology, Aretaieion Hospital, Medical School, National and Kapodistrian University of Athens, 76 Vasilissis Sofias Avenue, 11527 Athens, Greece

**Keywords:** vaginal delivery, caesarean section, delivery mode, stress, cortisol, growth hormone, interleukin 6, insulin-like growth factor 1

## Abstract

Background: How do stress related phenomena during labor differ between vaginal delivery (VD) and elective cesarean section (CS), remains of heightened interest. The purpose of this study is to investigate discrepancies regarding the stress response during VD and CS. Methods: Cortisol, interleukin 6 (IL-6), growth hormone (GH) and insulin-like growth factor 1 (IGF-1) levels from parturients’ peripheral blood were evaluated on three time-points, namely during the first stage of labor (TP1), two hours post labor (TP2) and 48 h post labor (TP3). Levels were also evaluated from the umbilical cord blood. A total of 50 women were enrolled in this prospective cohort study, with 24 and 26 subjected to CS and VD, respectively. Results: No statistically significant differences were observed between the two groups at TP1. Only GH levels presented the same pattern during the three time-points among both groups. In the umbilical cord blood, the CS group presented statistically significant higher IGF-1 and GH levels. In the umbilical cord blood, IGF-1 and GH levels were positively correlated, while GH and cortisol levels were negatively correlated. Conclusion: CS is a less stressful procedure than VD and is further associated with less intense inflammation, albeit with a longer inflammatory response period. Labor physiology during CS differs considerably regarding respective observations during VD. This merits extensive investigation in order to decipher these data for optimal clinical practice and guidelines.

## 1. Introduction

From a teleological point of view, stress response is a fundamental requirement for the survival of the human species. Threat, trauma, infection, surgery, labor, emotional disorders, depression, physical exercise, and malnourishment may all serve as stressor factors [1,2,3,4,5]. Interestingly, stressor factors may even impair the reproductive dynamics of the organisms [2,6]. A compromised reproductive potential due to stress has been acknowledged and may be managed through in vitro fertilization (IVF) treatment. Poor oocyte and sperm quality, as well as reduced fertilization and implantation rates and subsequent jeopardized pregnancy and obstetrics outcomes may be equally attributed to stress [7,8]. On the other hand, in regards to pregnancy and labor, studies indicate that stress-induced phenomena, mainly originating from the hypothalamic-pituitary-adrenal (HPA) axis, are essential for embryo development, as well as the preservation and the completion of pregnancy [9,10]. Research investigating the functionality of the HPA axis and stress response during pregnancy and labor may unravel valuable evidence in our quest for optimal management. Furthermore, our understanding of the pathways involved should be strengthened prior to suggesting any novel approach in the clinical practice.

Labor is considered an intense and stressful condition, both for the mother and for the fetus. At the terminal stages of pregnancy and as fetal development progresses, the intrauterine environment is inadequate to fully support further fetal survival. Hence, the fetus experiences a severe stressful stimuli [11]. Fetal cortisol induces Corticotropin Releasing Hormone (CRH) secretion from the placenta via a positive-feedback loop phenomenon [12]. During the terminal stage of pregnancy reaching labor time, the fetal HPA axis is strongly activated following a response to the stressful intrauterine environment, stimulating the secretion of cortisol and dehydroepiandrosterone sulfate (DHEAS) from the fetal adrenal glands [13]. DHEAS is converted to estrogens from the placenta’s aromatase, and, thus, a significant increase of estrogens levels is observed during vaginal delivery (VD). High estrogen levels in coordination with progesterone reduction promote uterine construction and the modification of connective tissue that allow cervical ripening and dilatation [14]. Following childbirth, CRH levels rapidly decrease to the basal levels at which they are observed prior to pregnancy. During labor, Adrenocorticotropic hormone (ACTH) reaches its maximum levels, and similarly to CRH, it is rapidly reduced back to the non-pregnant levels following childbearing [14]. 

All the aforementioned mechanisms refer to the physiology of VD. Nevertheless, nowadays, approximately one-third of births in the United States of America (USA) are performed via cesarean section (CS) delivery [15]. A similar trend has also been observed in several countries of high socioeconomic status, while others even report a significantly higher CS rate compared to the 15% recommended rate by the World Health Organization [16]. Among the countries of the Organization for Economic Co-Operation and Development (OECD), the CS rate has increased from 14.4% in 1990 to 25.8% in 2009, and this rise cannot be attributed to a possible equal rise of the obstetric risk factors. According to the National Institute for Health and Care Excellence (NICE) guidelines, delivery via CS is recommended under specific circumstances. Interestingly, CS is also recommended following maternal request on the grounds of maternal anxiety related to VD [17]. Despite the establishment of specific guidelines, recent studies demonstrate that, nowadays, a worldwide trend towards CS overuse is observed. This trend seems to be enabled by both maternity hospitals’ policies and clinicians’ routine practices. This may be driven by the overwhelming trend of elective CS. The option of elective CS is fueled by the desire of the parturient to avoid VD [18,19]. 

Despite the fact that CS is widely employed as a delivery method, further investigation on the topic of the physiology vis a vis the CS delivery is required, especially regarding the stress-related hormonal response during this practice [14]. There are indications supporting that both maternal and fetal HPA axis functionality is modified during CS in comparison to VD. From the maternal aspect, improvements regarding analgesia treatment during CS seem to reduce maternal stress, as indicated from the lower cortisol levels in peripheral blood samples obtained from mothers subjected to delivery via CS in comparison to those subjected to VD [20]. Regarding the infant, data provided are in the line of a reduced stress response regarding delivery via CS, as reflected by the reduced cortisol levels in blood obtained from the umbilical cord [21]. In addition, as suggested from studies performed on both humans and animal models, birth stress is strongly related to the long-term programming of the infant HPA axis [22]. Thus, studies investigating the determinants of stress physiology at birth and the relationship between maternal and fetal response to the stress during birth are of high significance. Such studies may define appropriate management regarding VD and CS for patients suffering from endocrine disorders such as adrenal insufficiency [23].

The aim of this study was to provide a comprehensive analysis of the stress related hormonal response during VD and elective CS through the evaluation of the levels of cortisol, interleukin 6 (IL-6), growth hormone (GH) and insulin-like growth factor 1 (IGF-1). Cortisol in coordination with catecholamines (epinephrine and norepinephrine), which are secreted when the locus coeruleus (LC) region is activated, regulate the “fight-or-flight” response and temporarily increase energy production [24]. Furthermore, cortisol reduces inflammation in the body and suppresses the immune system. Inflammation is a major stress factor, and inflammatory reactions mediate the production of pro-inflammatory cytokines such as IL-6. It has also been shown that IL-6 could initiate stress response phenomena via their receptors in the central nervous system [25,26]. Additionally, during the stress response, the production of plural hormones is modified. GH secretion is increased during and following operative treatment, and IGF-1 secretion is similarly increased following physical exercise. All these adaptations are considered to be of high significance, leading to the mobilization of energy sources while promoting the individuals’ adaptation to new conditions [27]. This work may open a new line of investigation, as it is the first report in the field aiming to fully examine and attempt respective associations all-inclusive of the aforementioned endocrine related events during either VD or elective CS. This may be ascertained by providing insight regarding the hormonal characteristics and how this profile may be adjusted and differentiated accordingly depending on delivery type.

## 2. Materials and Methods

This prospective observational study was carried out between 1 May, 2016 and 31 September, 2018 at the Department of Obstetrics and Gynecology, “Ippokrateio” General Hospital-Health Center of Kos, Kos Island, Greece, in collaboration with the Department of Physiology, Medical School, National and Kapodistrian University of Athens, Greece. The Hospital ethics board approved the study protocol and the consent form in accordance to the Helsinki declaration. All women who were eligible to participate in the study were included following oral and written consent.

### 2.1. Characteristics of Study Participants

The inclusion criteria for recruitment in the study were the following: Healthy women aged 20–43 years old with hitherto uncomplicated singleton pregnancies a complication-free general health medical record who underwent spontaneous VD or elective CS prior to labor onset, at a gestational age ranging from 37 to 40 weeks. The only indications for inclusion in the elective CS group were the following: Women presenting with previous CSs and parturients expressing the desire to avoid the prospect of pain related anxiety and distress that may be associated with VD. The exclusion criteria were the following: Women aged <20 years old or women aged >43 years old; multiple pregnancies; delivery prior to 37 or following 40 weeks of gestation; pregnancy complications, namely hypertension, gestational disorders or preeclampsia; pathological umbilical vein Doppler waveforms; known oligohydramnios or hydramnios; maternal conditions that could compromise fetal growth such as diabetes mellitus or gestational diabetes; maternal hypothalamus–pituitary–adrenal axes disorders; ongoing corticosteroid treatment; maternal comorbidities, namely autoimmune disorders and hepatic insufficiency; women who achieved pregnancy via assisted reproduction technology treatment; and women who presented with a medical history of complications in previous pregnancies. The sample size of this study (*n* = 50) was divided in two groups according to the type of delivery, namely the vaginal delivery group (VDG) (*n* = 26) and the caesarean section group (CSG) (*n* = 24). No complications including meconium-stained amniotic fluid, cardiotocograph abnormalities or pathological durations of the labor were reported during the delivery process in any of the study participants for both VDG and CSG groups. Both VDs and CSs were performed under epidural analgesia. 

### 2.2. Collection and Analysis of the Blood Samples

Maternal peripheral blood samples were collected from all study participants. Women were subjected to blood sampling from the median antebrachial vein at three different time-points. Time-Point 1 (TP1): Samples collected at the first stage of labor (cervical diameter < 6 cm) for VDG or 30 min following admission to the hospital for the CSG. Time-Point 2 (TP2): Samples collected 120 min following placenta delivery. Time-Point 3 (TP3): Samples collected 48 h following placenta delivery. Umbilical cord blood samples were collected following placenta delivery from the umbilical cord vein. 

The blood samples were collected in covered Ethylenediaminetetraacetic acid (EDTA) test tubes. Following collection, blood samples were left undisturbed at room temperature to clot for 30 min. The clots were removed by centrifuging the samples at 4000 rounds/min for 10 min in a refrigerated centrifuge. Following centrifugation, the supernatant (blood serum) was transferred into 0.5 mL aliquots and stored at −80 °C until further analysis. The blood serum concentrations of cortisol, interleukin-6 (IL-6), growth hormone (GH) and insulin-like growth factor 1 (IGF-1) were determined by employing standard competitive (for cortisol) or sandwich (for GH, IGF-1 and IL-6) enzyme-linked immunosorbent assay (ELISA) employing commercially available kits (human growth hormone Quantikine ELISA Kit for GH, human IGF-1 Quantikine ELISA Kit for IGF-1, human IL-6 Quantikine ELISA Kit for IL-6, and cortisol parameter assay kit for Cortisol, all provided by the R&D Systems Inc., Minneapolis, USA) according to the manufacturer’s instructions. Analysis was performed employing 96-well microtiter plates, and color formation was measured with a microplate reader (Varsamax, Molecular Devices, Sunnyvale, CA, USA) at 450 nm. A SoftMax Pro software (Molecular Devices) was employed in order to perform calculations. All samples were analyzed in duplicate, and the results presented hereby correspond to the respective average value.

### 2.3. Statistical Analyses

The VDG and the CSG groups were statistically compared to each other with respect to the levels of GH, IGF-1, cortisol and IL-6 at TP1, TP2 and TP3. Furthermore, statistical analysis was performed with regards to clinical data extracted from medical records, namely maternal age, gestational age, body mass index (BMI), parity, infants’ Apgar score (at 5 min), infants’ weight, and infants’ sex. All data analyses were performed using the R programming language for statistical purposes. Data are presented as average and standard deviation (SD) for normally distributed values. Regarding not normally distributed values median and range values are presented. Values lacking normal distribution were presented employing median and range values. The Shapiro–Wilk normality test was implemented in order to assess whether the tested data originated from a normally distributed population. Analyses were performed employing the parametric T-Test for normally distributed and skewed variables, and we also employed the non-parametric Mann–Whitney test for not normally distributed variables. Furthermore, in order to assess the possible effects of the type of delivery on GH, IGF-1, cortisol and IL-6 levels to both VDG and CSG, the mixed-model ANOVA and Tukey’s Honestly Significant Difference (HSD) was performed. Spearman’s correlation coefficient (*r*) was also performed to investigate all possible correlations between the investigated hormonal levels for both VDG and CSG. Confidence intervals of 95% were calculated for each variable, and a *p*-value < 0.05 was considered statistically significant. 

## 3. Results

A total of 50 women were enrolled in the present prospective study. Twenty-four of them delivered via CS and 26 delivered via VD. Maternal age, BMI, newborn’s weight, newborn’s sex, and the Apgar score did not differ with statistical significance between the two groups. The mean and SD of the above characteristics are presented in Table 1.

### 3.1. Between and within Group Differences

Between and within group differences were evaluated employing mixed methods ANOVA and pairwise comparison was performed via the Tukey HSD test. The type of labor was regarded as the between groups variance, and the time points were regarded as the within groups variance. Cortisol, IL-6 and IGF-1 presented with both between- and within-group differences. GH presented only with within group differences. The levels of the above-mentioned hormones and factors did not differ during the TP1. The levels of cortisol, IL-6, IGF-1 and GH at every time point are presented in Table 2. 

**Figure 1:**
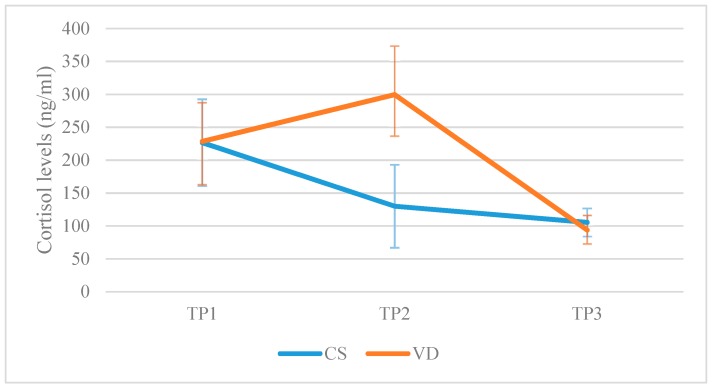
Cortisol levels reported in the caesarean section (CSG) group and in the vaginal delivery group (VDG), respectively, on each of the three separate time-points (TP1: Prior to labor; TP2: 120 min following placenta delivery; TP3: 48 h following placenta delivery).

Cortisol levels did not differ at TP3 between the VDG and the CSG. The CSG presented with lower levels compared to the VDG (129.93 ± 63.10 vs. 299.58 ± 74.00, *p*-value < 0.001) at TP2. In the CSG, cortisol levels dropped at TP2 compared to TP1 (Mean Difference (MD): −96.75, lower: −143.64, upper: −49.87, *p*-value < 0.001) but stabilized at TP3 (MD: −24.49, lower: −71.37, upper: 22.39, *p*-value = 0.66). Comparing TP1 and TP3, cortisol levels dropped (MD: −121.25, lower: −168.13, upper: −74.16, *p* < 0.001). In the VDG, cortisol levels raised at TP2 compared to TP1 (MD: 71.04, lower: 25.99, upper: 116.09, *p*-value < 0.001) but dropped at TP3 (MD: −205.77, lower: −250.82, upper: −160.73, *p*-value < 0.001). Comparing TP1 and TP3, cortisol levels were lower at TP3 (MD: −134.73, lower: −179.78, upper: −89.69, *p*-value < 0.001). A graphical representation is presented in Figure 1.

**Figure 2:**
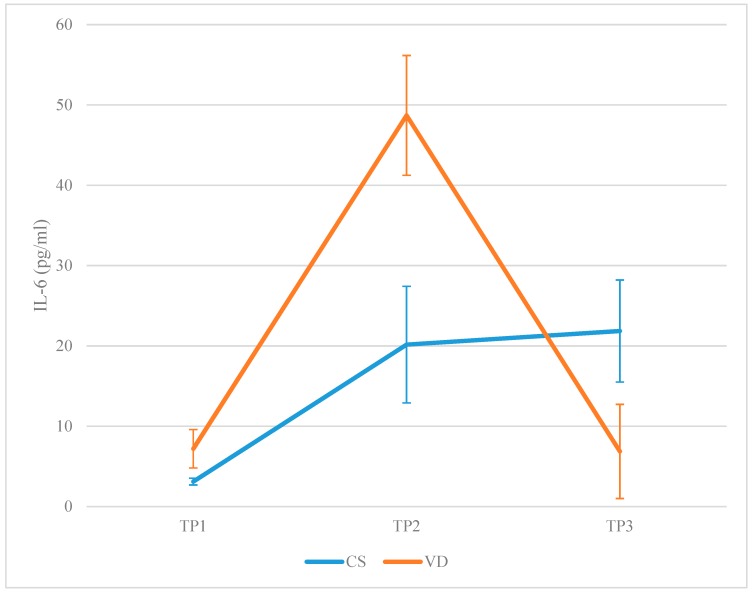
Interleukin-6 (IL-6) levels reported in the CSG and in the VDG, respectively, on each of the three separate time-points (TP1: Prior to labor; TP2: 120 min following placenta delivery; TP3: 48 h following placenta delivery).

IL-6 levels were lower in the CSG at TP2 (20.15 ± 7.25 vs. 48.70 ± 7.45, *p*-value < 0.001) and higher at TP3 (21.85 ± 6.35 vs. 6.86 ± 5.86, *p*-value < 0.001) compared to the VDG. In the CSG, IL-6 levels raised at TP2 (MD: 17.05, lower: 12.27, upper: 21.82, *p*-value < 0.001) and stabilized at TP3 (MD: 1.70, lower: −3.08, upper: 6.47, *p*-value = 0.91). Comparing TP1 and TP3, IL-6 levels were higher at TP3 (MD: 18.74, lower: 13.97, upper: 23.52, *p*-value < 0.001). In the VDG, IL-6 levels raised at TP2 (MD: 41.52, lower: 36.93, upper: 46.10, *p*-value < 0.001) and then dropped at TP3 (MD: −41.84, lower: −46.43, upper: −37.25, *p*-value < 0.001). Comparing TP1 and TP3, IL-6 levels did not differ (MD: −0.33, lower: −4.91, upper: 4.26, *p*-value = 0.99). A graphical representation is presented in Figure 2.

**Figure 3:**
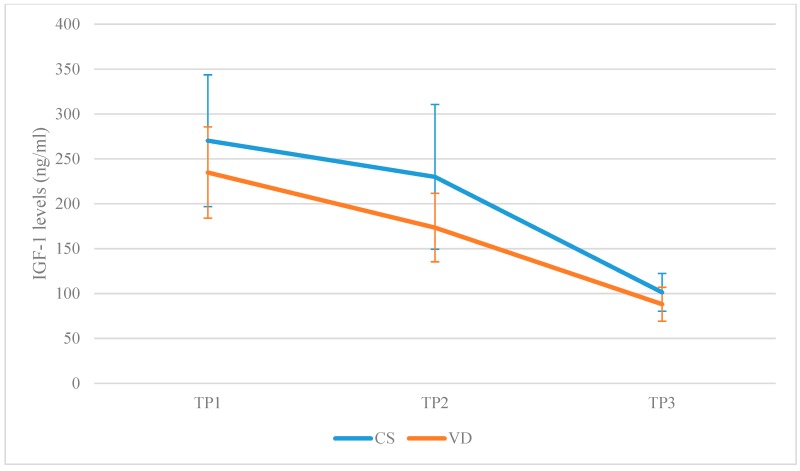
Insulin-like growth factor 1 (IGF-1 levels reported in the CSG and in the VDG, respectively, on each of the three separate time-points (TP1: Prior to labor; TP2: 120 min following placenta delivery; TP3: 48 h following placenta delivery).

IGF-1 levels were higher at TP2 in the CSG (230 ± 80.63 vs. 173.15 ± 38.12, *p*-value = 0.004) in comparison to VDG, but they were similar between the two groups at TP3 (101.38 ± 80.63 vs. 88.19 ± 18.89, *p*-value = 0.94). In the CSG, IGF-1 levels did not differ significantly between TP1 and TP2, although a trend was observed (MD: −42.00, lower: −85.95, upper: 1.95, *p*-value = 0.07) and dropped at TP3 (MD: −126.87, lower: −170.82, upper: −82.91, *p*-value < 0.001). Comparing TP1 and TP3, IGF-1 levels were lower at TP3 (MD: −168.87, lower: −212.82, upper: −124.92, *p*-value < 0.001). In the VDG, IGF-1 levels dropped at TP2 (MD: −61.28, lower: −103.51, upper: −19.05, *p*-value = 0.006) and continued dropping at TP3 (MD: −85.31, lower: −127.55, upper: −43.09, *p*-value < 0.001). Comparing TP1 and TP3, IGF-1 levels were lower at TP3 (MD: −146.60, lower: −188.83, upper: −104.37, *p*-value < 0.001). A graphical representation is presented in Figure 3.

**Figure 4:**
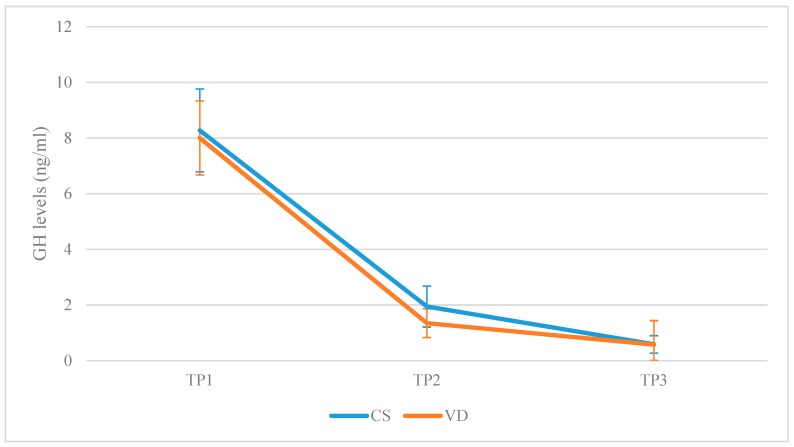
Growth hormone (GH) levels reported in the CSG and in the VDG, respectively, on each of the three separate time-points (TP1: Prior to labor; TP2: 120 min following placenta delivery; TP3: 48 h following placenta delivery).

In the CSG, GH levels were lower in TP2 compared to TP1 (MD: −6.33, lower: −7.15, upper: −5.50, *p*-value < 0.001) and continued dropping at TP3 (MD: −1.36, lower: −2.18, upper: −0.53, *p*-value < 0.001). Comparing TP1 and TP3, GH levels were lower at TP3 (MD: −7.69, lower: −8.51, upper: −6.86, *p*-value < 0.001). In the VDG, GH levels were lower in TP2 compared to TP1 (MD: −6.66, lower: −7.45, upper: −5.87, *p*-value < 0.001) but stabilized at TP3 (MD: −0.77, lower: −1.56, upper: 0.02, *p*-value = 0.62). Comparing TP1 and TP3, GH levels were lower at TP3 (MD: −7.43, lower: −8.22, upper: −6.63, *p*-value < 0.001). A graphical representation is presented in Figure 4.

### 3.2. Umbilical Cord Hormonal Levels

Cortisol and IL-6 levels in the umbilical blood did not present with a statistically significant difference between the two groups. IGF-1 and GH levels in the umbilical blood were statistically significantly higher in the CSG. The mean hormonal levels in the umbilical cord blood samples are presented in Table 3. In umbilical blood, IGF-1 and GH levels were positively correlated (*p*-value = 0.03), whereas cortisol and GH were negatively correlated (*p*-value = 0.01).

### 3.3. Correlation between Hormonal Levels, Maternal and Fetal Characteristics

Hormonal levels at TP1 and in the umbilical blood, maternal age and BMI, gestation week, newborn’s weight and Apgar score were assessed for possible correlations employing the Spearman’s correlation coefficient. In the total population, Apgar score was positively correlated with gestation week (*p*-value < 0.001), birth weight (*p*-value < 0.001) and IL-6 levels at TP1 (*p*-value = 0.02), and it negatively correlated with GH levels in the umbilical blood (*p*-value = 0.03). Birth weight was positively correlated with gestation week (*p*-value < 0.001) and IL-6 levels at TP1 (*p*-value = 0.007). The week of gestation was negatively correlated with GH levels in the umbilical cord (*p*-value = 0.03). 

Regarding possible correlations of the hormonal levels for each time points in the two groups, only IGF-1 and GH levels at TP3 were positively correlated in the VDG (*p*-value = 0.04).

## 4. Discussion

In the present prospective cohort study, the stress related hormonal response during labor via VD was compared to the labor via elective CS. The comparison was performed based on the evaluation of levels of cortisol, IL-6, GH and IGF-1 in healthy women with hitherto uncomplicated singleton pregnancies. In addition, in order to investigate the abovementioned response from the infants’ aspect, umbilical cord blood samples were collected immediately following placenta delivery. 

The statistical analysis revealed that in the VDG, cortisol levels in maternal peripheral blood increased immediately following labor, reaching the highest levels 2 h later, and then decreased, with the lowest levels of cortisol reported 48 h following VD. In contrast to the women who gave birth via VD, cortisol levels in women subjected to elective CS continuously decreased following labor. Forty-eight hours following labor, both groups presented with similar levels of cortisol in maternal peripheral blood. It is well documented that maternal serum cortisol levels increase markedly during pregnancy. A two-to-four fold rise in circulating cortisol, from 336 nmol/L (121.8 ng/mL) at the 16th week of gestation to 810 nmol/L (293.63 ng/mL) at the 38th week of gestation, has been reported [28]. During the third trimester, cortisol reaches the highest levels, marking approximately a three-fold increase in comparison to the non-pregnant state [10,29]. Even higher levels of circulating cortisol have been observed during the physical stress of VD. In a study published by Stjernholm et al. (2016), a comparison between VD and elective CS regarding maternal serum cortisol levels was performed. The authors concluded that VD is a significantly more stressful procedure in comparison to elective CS, as indicated from the higher serum cortisol levels observed in the VDG [23]. This observation was also documented in the study of Goldkrand et al. (1976) [30]. As a result, based on published data to date, labor via VD appears to be more stressful than CS. This is in agreement with the results provided in this study. At this point, it is of essence to highlight that in the present study, a statistically significant difference was observed between the studied groups regarding the gestational age at the time of the delivery. According to the results provided, women in the VDG gave birth on average at 38.54 weeks of gestation in comparison to those subjected to elective CS, who gave birth at 37.46 weeks of gestation. Taking into account the fact that the duration of pregnancy significantly affects circulating cortisol levels, this difference observed among the two groups could be viewed as a confounder. However, basal cortisol levels recorded immediately prior to labor (TP1) did not significantly differ between the two groups. Thus, we could safely assume that the difference observed between the two groups regarding the gestational age at the time of delivery did not serve as a confounder. This may be potentially attributed to the fact that this difference strictly referred to a single week during the later stages of term pregnancies. Studies in the field suggest that analgesia is a significant factor correlated with the reduced stress characterizing CS delivery. It is well documented that in the last decade, significant improvements have been described regarding analgesia treatment provided in women delivering via CS [23,31]. This observation has become of heightened clinical interest, particularly with regards to pregnant women with adrenal insufficiency. These patients are subjected to cortisol adjustment treatment, but the dosage protocol may be subject to modification prior to delivery, in order to ascertain a successful response towards labor stress. Moving forward from reports and in the presence of future robust data conclusively resulting to the verdict that women subjected to VD present with a higher degree of cortisol levels in comparison to CS, a dosage protocol adjusted to the type of labor may be established in the clinical practice. 

Evaluating the cortisol levels in the umbilical cord blood samples collected following VD and CS, no statistically significant difference was established between the two studied groups. Considering data provided from this study, the mode of the delivery, namely VD or elective CS, does not affect the infants’ cortisol levels. However, this observation is not supported by data available in literature to date. Thus far, there has been a limited number of published studies in which the relation between fetal cortisol levels and mode of the delivery has been investigated. Data provided from of these studies suggest that the mode of delivery as well as the type of analgesia employed during labor are significantly associated with fetal hormonal stress response, as indicated from the higher levels of catecholamines and cortisol observed in infants birthed via VD in comparison to those birthed via elective CS [20,21,32]. In a recent study published by Schuller et al. (2012), data corresponding to newborns delivered via different modes of labor was compared in regards to the stress response and the pain expression recorded. The authors concluded that newborns delivered via vaginal delivery are characterized by a higher incidence of stress response and pain expression in comparison to those delivered via CS [33]. Interestingly, it has been voiced that stress experienced by infants during the different modes of labor could significantly affect the function and the maturation of the HPA axis in newborns’ later life [32,34]. Despite the fact that this constitutes an observation of high significance, a limitation in safely extracting conclusions is attributed to the small number of studies indicating this relationship. Thus, a number of larger observational studies may be required in order to provide higher quality evidence. It is imperative for the scientific community to be driven towards large well-designed studies with the main outcome measures focusing on pediatric data which identifies possible associations between mode of delivery and HPA axis maturation during the neonatal period. In light of this, it should be noted that for this study’s participants, the authors observed no complications during delivery or any signs of fetal distress indicated by meconium stained amniotic fluid. In addition, all newborns in both VDG and CSG presented with an Apgar score >7. The evaluation of such parameters is of underling significance, as fetal distress may present as a confounder that considerably affects cortisol levels in newborns.

It has been voiced that the physical stress the parturient experiences during VD activates the cytokines’ production network, leading to the secretion and elevation of cytokines levels on maternal serum. These cytokines manage to reach the newborns’ peripheral circulation by crossing the placenta. These levels stand as the first line of defense towards managing pathogens and infection during the late neonatal period [35]. It is well documented that inflammation is a major stressor factor, and inflammatory reactions mediate the production of pro-inflammatory cytokines such as IL-6. It has also been reported that these cytokines, such as IL-6, per se could initiate stress response phenomena via their receptors in the central nervous system (CNS). In order to investigate the maternal stress response in correlation to the delivery mode, the evaluation of IL-6 levels in maternal peripheral blood was deemed necessary. To our knowledge, only a small number of studies have evaluated IL-6 levels in maternal peripheral blood between women subjected to VD in comparison to women subjected to delivery via elective CS. In a study published by Mojaveri et al. (2014), women subjected to CS presented with lower levels of IL-6 in comparison to those delivering via VD [36]. Similar results were also provided from other studies in the field [37,38,39]. The present study’s results are concordant to the vast majority of the studies, indicating that, in both VDG and CSG, IL-6 increases following labor. However, in the VDG, the two-fold rise of the IL-6 observed 120 min following placenta delivery, in comparison to the CSG, indicates a more intense inflammatory response in the VDG. Interestingly, 48 h following placenta delivery, IL-6 levels decreased to the levels reported prior to labor in the VDG. In contrast, in the CSG, IL-6 levels did not decrease 48 h following placenta delivery—they remained in the same levels as those observed 120 min following labor. Further to that, a statistical analysis revealed that Apgar score, as well as birth weight, were positively correlated with IL-6 levels at the initial stages of labor, indicating that the presence of pro-inflammatory cytokines is an essential component for an optimal outcome during labor. Interestingly, it has been reported that high IL-6 levels 24 h post-operatively are associated with a high risk of complications [40]. In conclusion, regarding IL-6 levels in maternal peripheral blood samples, available results from published studies, which are in agreement with the results provided herein, indicate that women subjected to CS experience a less intense inflammation but a longer inflammatory response period, in comparison to women subjected to VD labor. This observation may be related to the fact that women undergoing a CS delivery present with a longer recovery period along with a prolonged stay in the maternity unit post-partum in comparison to women subjected to VD. High quality evidence, which should be provided from larger observational studies in the future, are essential in order to verify this significant observation.

Evaluating the IL-6 levels in the umbilical cord blood samples collected following VD and CS, respectively, no statistically significant difference was established between the two studied groups. Considering data provided from this study, the mode of the delivery does not appear to affect infants’ IL-6 levels. To our knowledge, there is a limited number of published studies investigating the relation between fetal IL-6 levels and mode of the delivery, and the results provided are controversial. Some studies indicate that IL-6 levels in the umbilical cord of infants delivered via CS were significantly lower in comparison to respective levels observed in infants delivered via VD [36,39]. However, it has also been suggested that CS delivery is associated with higher IL-6 levels in the umbilical cord blood in comparison to VD [41]. Interestingly, no relationship between the mode of delivery and IL-6 levels in the umbilical cord was indicated in the studies published by Takahashi et al. (2010), Fukuda et al. (2002), and De Jongh et al. (1999) [42,43,44]. Taking into account published data, it appears that the questions of “whether, how, and to what extent delivery mode influences concentration of pro-inflammatory cytokines such as IL-6 in neonates” remain unanswered. Future studies exploring the possible correlation between the mode of delivery and IL-6 levels in infants’ circulation merit investigation in future studies.

It is well documented that during acute stress, the secretion of GH and prolactin is upregulated. Concurrently, GH promotes the secretion of liver IGF-1. Taking into account that labor results to acute stress, the authors set out to investigate whether VD and CS play a role in affecting the secretion levels of these hormones. Regarding GH levels in the maternal peripheral blood, no statistically significant difference could be established between the two groups in any of the time-points evaluated. In both groups, GH levels decreased following labor. Interestingly, in the CSG, a statistically significant reduction regarding GH levels was established in the time-frame of 2 h and 48 h post labor. Despite the fact that a similar reduction was also observed in the VDG regarding GH levels in the above mentioned time-frame, this was not statistically significant. However, in both groups, GH reached the lowest levels 48 h following placenta delivery. A similar trend to GH levels was also observed regarding IGF-1 levels. In both VDG, and CSG, IGF-1 levels decreased continuously following placenta delivery. However, in the CSG, the reduction observed regarding IGF-1 levels in the time-frame of 2 h and 48 h post labor was not statistically significant marginally, probably due to the small number of participants included in the study (*N* = 24 in the CSG). In addition, IGF-1 levels in the CSG presented to be higher in comparison to IGF-1 levels in the VDG 2 h following labor. Though women in the VDG presented with higher levels of cortisol and IL-6 immediately following delivery indicating a heightened stress response, results show that GH and IGF-1 levels dropped equally immediately following delivery. It is well known that during gestation, both GH and IGF-1 increase progressively. This increase is mostly attributed to production of placental GH being homologous to the pituitary GH. In fact, following the 20th week of gestation, placental GH stands as the principal form of GH inhibiting the production of pituitary GH. The observation that the levels of GH and IGF-1 reduce following delivery may be attributed to the placental delivery and the subsequent termination of placental GH secretion into the maternal circulation. Noting the drop regarding GH and IGF-1 levels in both delivery groups, it appears that the decrease was heightened in the VDG within the first 120 min following placenta delivery. This may be attributed to the fact that CS may serve as an inhibitor with respect to the reduction of hormonal levels in the CSG. Forty-eight hours following delivery, the levels of GH and IGF-1 appeared to be positively correlated and reached minimum levels, thus indicating that the function of the maternal GH axis had not been activated yet. This observation is of valued clinical interest with respect to special pregnant women categories such as those featuring GH insufficiency, as it may serve as an indication regarding the optimal timing in embarking on GH replacement therapy. To our knowledge, this is the first study in which the association between maternal plasma GH levels and type of delivery has been investigated. This conclusion should be further explored and supported by solid data, as its potential is promising.

Evaluating GH and IGF-1 levels in the umbilical cord blood samples collected following VD and CS, a statistically significant increased level regarding both hormones was observed in the CSG compared to the VDG. Taking into consideration that the levels of cortisol and IL-6 in the umbilical cord did not differ between the two groups of the study, the observed difference regarding GH and IGF-1 levels cannot be safely interpreted employing the stress response justification. Statistical analysis revealed a negative correlation between GH levels in the umbilical cord and gestational week. In addition to that, a positive correlation between GH levels and IGF-1 levels in the umbilical cord was observed. The difference between levels of GH and IGF-1 among the two groups may be attributed to the fact that women of the CSG gave birth one week earlier in comparison to the respective participants of the VDG. Additionally, given the positive correlation between Apgar score and gestational week, along with the negative correlation between GH levels and gestational week, the negative correlation between GH levels in umbilical cord blood and Apgar score may be explained. Though the function of GH axis does not appear to be related to labor stress, the extent to which the CS procedure may influence—through various mechanisms—the neonatal axis merits investigation. To our knowledge, only a single published study has explored the factors affecting GH axis during birth. This study indicates that levels of GH and IGF-1 following labor are directly associated with anthropometric characteristics and the sex of the neonate but not delivery mode [45]. Respective associations in the present study were not identified, possibly due to the small size of the studied population. Despite the fact that the GH–IGF axis does not appear to be related to labor stress, the present study indicates that levels of GH and cortisol in umbilical cord blood present with a negative correlation in the general population. In case our findings are confirmed by future studies and in light of the fact that cortisol may downregulate GH secretion from the pituitary [46], our observation could serve as a valid explanation of the elevated GH levels in the umbilical cord for neonates born via CS, as their cortisol levels appear reduced. In light of the fact that GH exerts an important influence during early neonatal development, an investigation of the effects of delivery mode on the neonates’ GH axis functionality presents with increased pediatric interest. Published data on animal studies concur that increased GH levels are associated with dysfunction of the adipose tissue and increased weight during the first three-to-six months [47]. Further to that, children born via CS tend to present with an increased weight in comparison to VD neonates [48]. Whether this phenomenon may be attributed to elevated GH levels in umbilical cord blood of elective CS neonates remains to be evaluated by future studies.

Data provided from the present study as well as similar data provided from other studies in the field indicate that the physiology of labor is subject to various adjustments when comparing elective CS to VD. Investigating these differences is of a high significance, not only from the aspect of basic research but most importantly in order to assist clinicians to make safe conclusions and practice the optimal management of the parturient. The impact of delivery mode on both the mother and infant merits in depth investigation through studying physiological processes with regards to perinatal and neonatal outcomes. During the last few decades, labor via elective CS has been observed as a worldwide trend, especially in countries of high social–economic status. Despite the fact that this trend seems to be promoted by both maternity hospitals’ policies and clinicians’ routine practices, our knowledge regarding CS physiology still remains limited, especially in the context of stress response during labor for both mothers and newborns. Studies in this field are limited, and the majority investigate stress response related phenomena individually, failing to depict stress-responses in an all-inclusive fashion. This is another reason that makes this study timely and essential, especially if we consider that birth stress is strongly related to the long-term programming of the infant HPA axis [22]. At this point, it is appropriate to state that the current study does not aim to dictate the practitioners’ options with regards to VD and CS. In clinical practice, labor via CS is recommended according to current guidelines as the gold standard delivery mode, under specific circumstances, including multiple pregnancies, breech presentation at term, placenta praevia and morbidly adherent placenta, as well as in order to prevent the transmission of maternal infections to infants. Nonetheless, in routine practice, clinicians may face several dilemmas regarding the appropriate management of women presenting with no indications for CS but wishing to deliver via CS on the grounds of anxiety related to VD. Under this prism, the present study aims to raise the practitioners’ awareness regarding stress related hormonal response during VD and elective CS, as well as the possible impact of stress physiology on both the mother and infant. Advanced knowledge on physiological process when CS may be an option based on desire should surely be of importance in decision making—equally so for the clinician and for the parturient, ascertaining an all-inclusive informed consent. 

## 5. Conclusions

The rationale of the present research was to examine and attempt respective associations between cortisol, IL-6, GH, and IGF-1 levels during either VD or elective CS in an effort to provide evidence regarding stress related hormonal response during VD and elective CS. Data presented herein indicate that CS is a significantly less stressful procedure for mothers in comparison to VD, and it is further associated with less intense inflammation, albeit with a longer inflammatory response period. From the infants’ perspective, GH and IGF-1 appear to be increased in the umbilical cord blood of CS born neonates. Nonetheless, whether this may be attributed to the mode of delivery remains unanswered. Even though this study interestingly provides data with significant perinatal and pediatric interest, its limitations—the small size of the study’s recruited participants—should be underlined. Data presented herein, along with previously published data, strengthen the idea that labor physiology during CS differs vastly considering respective observations during VD. Consequences related to these differences in regards to both the mother and child remain unidentified, and future large observational studies are required. The clinical end-point of the present study is that, until research reveals the holy grail of optimal practice, clinicians are bound to abide by current guidelines to avoid CS overuse and to evaluate each case management in the era of personalized medicine, considering that the physiological response during CS is significantly differentiated in comparison to VD.

## Figures and Tables

**Table 1:** Mean and standard deviation (Mean ± SD) of patients’ general characteristics.

Patients’ Characteristics	Vaginal Delivery Group (Mean ± SD)	Caesarean Section Group (Mean ± SD)	*p*-Value *
Maternal Age	29.69 ± 5.28	31.17 ± 5.28	0.34
Maternal Body Mass Index	26.77 ± 2.05	26.38 ± 2.32	0.53
Weeks of Gestation	38.54 ± 0.93	37.46 ± 1.08	0.001
Newborns’ Weight	3267.69 ± 354.65	3027.92 ± 511.69	0.06
Apgar Score	9.35 ± 1.07	8.88 ± 1.09	0.13
Newborns’ Sex Ratio (F/M)	13/13	8/16	0.26

*: *p*-value < 0.05 was considered statistically significant.

**Table 2:** Mean maternal serum hormonal levels reported on each of the three separate time-points (TP1: Prior to labor; TP2: 120 min following placenta delivery; TP3: 48 h following placenta delivery). Data are presented in the Mean ± SD format.

	Time Point 1		Time Point 2		Time Point 3	
	Vaginal Delivery Group	Caesarean Section Group	Vaginal Delivery Group	Caesarean Section Group	Vaginal Delivery Group	Caesarean Delivery Group
Cortisol	228.54 ± 58.71	226.68 ± 65.89	299.58 ± 74	129.93 ± 63.1	93.8 ± 22.2	105.44 ± 21.15
IL-6	7.19 ± 2.39	3.11 ± 0.43	48.7 ± 7.45	20.16 ± 7.25	6.86 ± 5.86	21.85 ± 6.35
IGF-1	234.79 ± 50.86	270.25 ± 73.4	173.51 ± 38.12	230 ± 80.63	88.19 ± 18.89	101.38 ± 21.07
GH	8 ± 1.33	8.28 ± 1.49	1.35 ± 0.51	1.95 ± 0.93	0.58 ± 0.87	0.59 ± 0.31

**Table 3:** Mean umbilical cord hormonal levels reported for the vaginal delivery and cesarean section group.

	Vaginal Delivery Group	Caesarean Section Group	*p*-Value
Cortisol	66.34 ± 19.22	60.25 ± 8.7	0.16
IL-6	5.15 ± 7.6	2.93 ± 0.49	0.09
IGF-1	54.98 ± 9.11	64.3 ± 14.27	0.01
GH	6.45 ± 1.06	9.27 ± 3.63	0.001

*: *p*-value < 0.05 was considered statistically significant. Data are presented in the Mean ± SD format.
